# RELATIONSHIP BETWEEN JOB STRESS AND BURNOUT AMONG KOREAN WORKERS IN SMALL AND MEDIUM-SIZED ENTERPRISES

**DOI:** 10.13075/ijomeh.1896.02650

**Published:** 2025

**Authors:** Ji-Hoon Kim, Shin-Goo Park, Seong-Cheol Yang, Hwan-Cheol Kim, Sang-Hee Hwang

**Affiliations:** 1 Inha University College of Medicine, Department of Occupational and Environmental Medicine, Incheon, South Korea; 2 Keimyung University College of Medicine, Department of Dentistry, Daegu, South Korea

**Keywords:** ICD-11, Korean, workers, job stress, burnout syndrome, small and medium-sized enterprises

## Abstract

**Objectives:**

Burnout is a work-related syndrome with growing relevance in occupational health. This study explored the association between job stress factors and burnout in Korean workers at small- and medium-sized enterprises (SMEs).

**Material and Methods:**

A total of 1024 employees from SMEs (50–299 workers) receiving outsourced occupational health services completed a cross-sectional survey. Job stress was measured using the *Korean Occupational Stress Scale* short form (KOSS), and burnout was assessed with the Korean version *Burnout Syndrome Scale* (KBOSS), aligned with International Classification of Diseases, 11th Revision (ICD-11) criteria. Burnout was evaluated across 3 dimensions: exhaustion, cynicism, and reduced efficacy. Burnout syndrome was defined as meeting all 3 dimensions. Multiple logistic regression analyses were performed to assess the associations between job stress factors and burnout.

**Results:**

Burnout syndrome was found in 3.3% of participants. Key findings with statistical measures include: 1) burnout syndrome association – high job demand (Q3: OR = 12.62, 95% CI: 2.03–78.41, p < 0.05) and high overall job stress (Q4: OR = 17.56, 95% CI: 1.40–220.76, p < 0.05); 2) exhaustion predictors – high job demand (Q3: OR = 10.71, 95% CI: 3.64–31.48, p < 0.001), inadequate compensation (Q4: OR = 2.06, 95% CI: 1.02–4.16, p < 0.05), and poor workplace culture (Q4: OR = 2.63, 95% CI: 1.11–6.24, p < 0.05); 3) paradoxical findings – low job autonomy associated with reduced exhaustion (Q4: OR = 0.23, 95% CI: 0.11–0.48, p < 0.001).

**Conclusions:**

Specific job stressors differentially impact burnout dimensions in Korean SMEs: Job demand and overall stress critically predict burnout syndrome, while inadequate compensation and poor workplace culture significantly affect exhaustion. The counterintuitive protective effects of reduced autonomy warrant further investigation. Organizations should prioritize evidence-based workload management and compensation fairness aligned with ICD-11 diagnostic patterns.

## Highlights

- Burnout assessed using ICD-11-based *Korean Burnout Syndrome Scale.*
- High job demand and overall stress are strongly linked to burnout syndrome.
- Each burnout dimension showed distinct links to job stress factors.

## INTRODUCTION

Work-related stress and psychosocial risks are among the most pressing concerns, not only for workers' health but also for workplace safety. These issues can have far-reaching consequences beyond the well-being of individual employees, affecting organizations, workplaces, and national economies [1]. Work-related stress can lead to mental health problems, such as anxiety, depression, fatigue, and substance abuse, as well as somatic symptoms, including cardiovascular diseases and gastrointestinal disorders [2]. Prolonged and persistent job stress can cause burnout, leading to decreased productivity and adversely affecting individuals' psychological and physical health [3].

Previous research has extensively documented the relationship between job stress and employee well-being across various cultural contexts. International studies have established that workplace stressors – including excessive workload, role ambiguity, lack of autonomy, and poor social support – significantly contribute to burnout development [4,5]. However, the specific manifestation and prevalence of these relationships vary considerably across different cultural and organizational environments.

Recent research on Korean workers has revealed unique characteristics of occupational stress. A high level of occupational stress may be associated with a high level of burnout, which in turn, leads to a high level of depression among Korean employees, with cultural factors playing a moderating role [6]. Studies during the COVID-19 pandemic found that 84.5% of Korean healthcare workers showed clinical exhaustion and 91.1% showed clinical disengagement, highlighting the severity of burnout in Korean work environments [7]. Furthermore, workplace stressors, such as long hours, job insecurity, and high-performance demands, have been associated with higher levels of depressive symptoms, anxiety, and burnout specifically among young Korean workers in metropolitan areas [8].

Despite extensive research, significant gaps remain in understanding the specific job stress factors that contribute to different dimensions of burnout within Korean organizational contexts. Most existing studies have focused on healthcare workers or general employee populations without systematically analyzing the differential effects of various job stressors on the 3 distinct burnout dimensions as defined by International Classification of Diseases, 11th Revision (ICD-11) criteria.

The conceptualization of burnout has undergone significant evolution in international health classifications. Under ICD-10 (1992), burnout was classified merely as a “state of burnout” within life management difficulties, considered a social problem rather than a health condition requiring systematic intervention [9].

The ICD-11 revision, effective from January 1, 2022, fundamentally transformed this understanding. Burnout is now categorized as a “syndrome” that results from “chronic workplace stress that has not been successfully managed” and is specifically recognized as an occupational phenomenon rather than a personal inadequacy [10]. The new ICD-11 definition includes 3 specific signs of workplace burnout:

– emotional exhaustion (extreme energy depletion and fatigue),
– cynicism and work-related negativity (detachment from and negative feelings toward one's occupation),
– reduced efficacy (negative evaluation of one's work performance and capabilities).

This redefinition emphasizes workplace responsibility for burnout prevention and management, shifting focus from individual resilience to organizational intervention strategies.

The Korean work environment presents unique challenges characterized by intense work culture, hierarchical organizational structures, and high-performance [11]. Several factors, such as long working hours, employment instability due to non-regular and contract employment, low compensation systems, and conflicts in interpersonal relationships within the workplace, have been identified as major factors that increase the risk of burnout among Korean workers [12]. Recent Korean studies have developed culturally adapted measurement tools, including the Korean version *Burnout Syndrome Scale* (KBOSS) based on ICD-11 criteria, a validated instrument specifically designed to measure the 3 ICD-11 dimensions of burnout in Korean workers [13]. However, systematic analysis linking specific job stress factors measured by validated Korean instruments to distinct burnout dimensions remains limited.

This study addresses critical knowledge gaps by providing the first comprehensive analysis of job stress-burnout relationships using both the *Korean Occupational Stress Scale* (KOSS) and KBOSS within Korean organizational contexts. Unlike previous studies that treated burnout as a unidimensional construct, this research examines differential relationships between specific job stressors and each of the 3 burnout dimensions (emotional exhaustion, cynicism, and reduced efficacy).

The novelty of this research lies in:

– systematic application of ICD-11 burnout criteria within Korean occupational settings;
– comprehensive examination of 7 distinct job stress domains (job demand, job autonomy, interpersonal conflict, job insecurity, organizational system, inadequate compensation, and workplace culture) and their differential effects on burnout dimensions;
– provision of empirical evidence for targeted intervention strategies based on stress-burnout pathway analysis;
– contribution to culturally-informed burnout prevention policies in Korean workplaces.

Based on the literature review and theoretical framework, this study tests the following hypotheses:

– H1: Job stress factors will show significant positive correlations with all 3 burnout dimensions (emotional exhaustion, cynicism, and reduced efficacy);
– H2: Different job stress domains will demonstrate significant associations with distinct burnout dimensions, with workload and role stress showing positive associations with emotional exhaustion;
– H3: Organizational factors (organizational climate, inadequate compensation) will demonstrate significant positive relationships with the cynicism dimension of burnout;
– H4: Job insecurity and lack of control will show significant associations with reduced efficacy dimension of burnout;
– H5: The combined effect of multiple job stress factors will explain substantial variance in burnout syndrome (BOS) beyond individual stress domain contributions.

This study aims to contribute to evidence-based policy development and intervention strategies for burnout prevention by:

– identifying job stress factors with the greatest impact on each burnout dimension,
– providing empirical foundation for workplace intervention priorities,
– informing organizational policies for stress management and burnout prevention,
– advancing understanding of culture-specific stress-burnout relationships in Korean work environments.

The findings will support development of targeted prevention programs and inform occupational health policies aligned with ICD-11 burnout classification standards.

## MATERIAL AND METHODS

### Participants

This cross-sectional study was conducted among workers employed in small- and medium-sized enterprises (SMEs) with 50–299 employees receiving outsourced occupational health management services. The study protocol was approved by the Institutional Review Board of Inha University Hospital in South Korea (No. 2025-03-025-000), and all participants provided written informed consent before participation.

#### Recruitment and data collection

Participants were recruited through occupational health service providers in May 2023 – January 2025. The inclusion criteria were:

– full-time employees aged ≥19 years,
– employed for ≥3 months in their current position,
– able to understand and complete the Korean-language questionnaire.

Exclusion criteria included:

– temporary or contract workers,
– workers on extended leave,
– incomplete questionnaire responses (missing >20% of items).

Among 2060 workers initially approached, 1105 provided complete responses (response rate: 53.6%). Participants with missing values >20% were excluded using listwise deletion, resulting in a final analytical sample of 1024 participants. The authors acknowledge that listwise deletion is not optimal, but due to the requirements of standardized national reference values (Korea Occupational Safety and Health Agency [KOSHA] quartiles), complete data were necessary for categorization.

### Measures

#### Job stress assessment

Job stress was measured using the short form of KOSS, consisting of 24 items [14]. The KOSS evaluates 7 dimensions of job stress:

– job demand (4 items),
– insufficient job autonomy (4 items),
– interpersonal conflict (3 items),
– job insecurity (2 items),
– organizational system (4 items),
– inadequate compensation (3 items),
– workplace culture (4 items).

This instrument was specifically developed and validated for the Korean work environment, demonstrating high reliability (Cronbach's α ≥0.7) and validity in previous studies.

Importantly, job stress scores were categorized into quartiles (Q1–Q4) based on the standardized reference values established by the KOSHA [15]. This categorization is not arbitrary but follows nationally recognized standards for occupational stress assessment in Korea. However, in the case of the relationship conflict domain, Q1 and Q2 were combined due to the absence of burnout cases in Q1, which would have led to overestimation of risk ratios. The internal consistency of the KOSS subscales was assessed using Cronbach's α, with all subscales showing acceptable reliability (α >0.70) (Table 1).

**Table 1.** Internal consistency of measurement instruments among workers at small- and medium-sized enterprises (N = 1024), South Korea, May 2023 – January 2025

Domain	Items [n]	Cronbach's α	Score [pts] (M±SD)
*Korean Occupational Stress Scale* (KOSS)			
job demand	4	0.789	2.457±0.346
insufficient job autonomy	4	0.709	2.683±0.122
interpersonal conflict	3	0.732	2.003±0.172
job insecurity	2	0.737	2.107±0.023
organizational system	4	0.787	2.362±0.229
inadequate compensation	3	0.761	2.455±0.335
workplace culture	4	0.716	2.042±0.508
*Korean Burnout Syndrome Scale* (KBOSS)			
emotional exhaustion	4	0.945	3.758±0.420
cynicism	4	0.849	3.034±0.712
reduced efficacy	4	0.831	4.820±0.084

All Cronbach's α values exceeded the acceptable threshold of 0.70.

#### Burnout assessment

Burnout was assessed using KBOSS, a 12-item instrument. The KBOSS measures 3 dimensions of burnout:

– emotional exhaustion – psychological and physical fatigue due to work (4 items),
– cynicism – negative attitude and detachment from one's job (4 items),
– reduced efficacy – negative perception of one's work performance and ability (4 items).

This scale was specifically developed to reflect Korean organizational culture characteristics such as long working hours and hierarchical decision-making structures, making it more culturally appropriate than Western-developed scales like the *Maslach Burnout Inventory* (MBI). The internal consistency reliability (Cronbach's α) of the KBOSS was excellent, with values of 0.916 for emotional exhaustion, 0.865 for cynicism, 0.819 for reduced professional efficacy, and 0.813 for the total scale. The KBOSS aligns with the ICD-11 definition of BOS. The internal consistency of the KBOSS subscales was assessed using Cronbach's α, with all subscales showing acceptable reliability (α > 0.70) (Table 1).

### Variables and operational definitions

#### Dependent variables

For the KBOSS, participants were classified as having burnout in each dimension if they exceeded the established cutoff scores. Burnout syndrome was defined as meeting the cutoff criteria across all 3 dimensions simultaneously.

#### Independent variables

Job stress factors were analyzed both as individual dimensions and as a total score. Each dimension was categorized according to the KOSHA's quartile reference values, with Q1 representing low stress, Q2 low-moderate stress, Q3 high-moderate stress, and Q4 high stress.

#### Control variables

The following variables were included as potential confounders based on previous literature: sex, job type (production work, office work, service work), shiftwork status (yes, no) [16], marital status (yes, no), and work experience [17] (<1 year, 1–5 years, 5–10 years, and >10 years).

### Statistical analysis

#### Descriptive analysis

Participant characteristics were examined using descriptive statistics. Continuous variables were presented as means (M) and standard deviations (SD), while categorical variables were presented as frequencies and percentages.

#### Main analysis

The association between job stress factors and burnout was analyzed in 3 steps:

1. univariate analysis – χ^2^ tests were used to examine the basic associations between job stress factors and BOS;
2. dimension-specific analysis – multiple logistic regression analyses were conducted to examine the impact of each job stress factor and total job stress score on the 3 dimensions of burnout (exhaustion, cynicism, and inefficacy), adjusting for all control variables;
3. burnout syndrome analysis – multiple logistic regression analysis was performed to analyze the effects of job stress factors on workers diagnosed with BOS, adjusting for control variables.

All results are presented as odds ratios (ORs) with 95% confidence intervals (CIs). Statistical analyses were performed using SPSS v. 25.0 (IBM Corp., Chicago, IL, USA), with statistical significance set at p < 0.05. The decision to use categorical analysis rather than continuous variables or structural equation modeling was based on the practical application of nationally standardized occupational stress assessment criteria, which facilitates direct implementation in occupational health practice.

#### Construct validity assessment

To ensure the structural validity of this study's measurement instruments, confirmatory factor analysis (CFA) was conducted using structural equation modeling. The KOSS 7-factor model and KBOSS 3-factor model were tested separately using maximum likelihood estimation. Model fit was evaluated using multiple indices: χ^2^/df ratio (<3.0), comparative fit index (CFI >0.90), Tucker-Lewis index (TLI >0.90), root mean square error of approximation (RMSEA <0.08), and standardized root mean square residual (SRMR <0.08). Factor loadings ≥0.70 were considered acceptable for construct validity.

## RESULTS

### Measurement model validation

Prior to hypothesis testing, confirmatory factor analysis was conducted to validate the factor structure of both measurement instruments. For the KOSS, the 7-factor model demonstrated acceptable fit indices: χ^2^(246) = 895.42, p <0.001, CFI = 0.941, TLI = 0.932, RMSEA = 0.045 (90% CI: 0.042–0.048), SRMR = 0.043. All factor loadings were in the range of 0.70–0.83, indicating adequate construct validity (Table 2). The KBOSS 3-factor model showed an excellent fit: χ^2^(51) = 285.36, p <0.001, CFI = 0.971, TLI = 0.963, RMSEA = 0.038 (90% CI: 0.034–0.042), SRMR = 0.032. Factor loadings ranged 0.71–0.83, confirming the 3-dimensional structure of burnout (Table 2). Both models met recommended criteria for structural validity, supporting the use of these instruments in subsequent analyses (Table 3).

**Table 2.** Confirmatory factor analysis results for the Korean Occupational Stress Scale (KOSS) and Korean Burnout Syndrome Scale (KBOSS) models among workers at small- and medium-sized enterprises (N = 1024), South Korea, May 2023 – January 2025

Variable	Standardized estimate	SE	t	p
KOSS^a^				
Job demand				
KOSS_Q1	0.74	0.03	24.68	<0.001
KOSS_Q2	0.71	0.03	23.67	<0.001
KOSS_Q3	0.78	0.02	26.12	<0.001
KOSS_Q4	0.73	0.03	24.32	<0.001
Job autonomy				
KOSS_Q5	0.72	0.03	24.01	<0.001
KOSS_Q6	0.76	0.02	25.34	<0.001
KOSS_Q7	0.73	0.03	24.45	<0.001
KOSS_Q8	0.70	0.03	23.33	<0.001
Relationship conflict				
KOSS_Q9	0.77	0.03	25.89	<0.001
KOSS_Q10	0.74	0.03	24.78	<0.001
KOSS_Q11	0.79	0.02	26.45	<0.001
Job insecurity				
KOSS_Q12	0.83	0.02	27.91	<0.001
KOSS_Q13	0.81	0.02	27.12	<0.001
Organizational system				
KOSS_Q14	0.78	0.02	26.23	<0.001
KOSS_Q15	0.75	0.03	25.17	<0.001
KOSS_Q16	0.80	0.02	27.34	<0.001
KOSS_Q17	0.77	0.03	25.98	<0.001
Inadequate compensation				
KOSS_Q18	0.74	0.03	24.89	<0.001
KOSS_Q19	0.72	0.03	24.12	<0.001
KOSS_Q20	0.76	0.02	25.67	<0.001
Workplace culture				
KOSS_Q21	0.79	0.02	26.78	<0.001
KOSS_Q22	0.77	0.03	25.94	<0.001
KOSS_Q23	0.81	0.02	27.45	<0.001
KOSS_Q24	0.78	0.02	26.32	<0.001
KBOSS^b^				
Exhaustion				
KBOSS_Q1	0.81	0.02	27.15	<0.001
KBOSS_Q2	0.78	0.02	26.03	<0.001
KBOSS_Q3	0.83	0.02	28.42	<0.001
KBOSS_Q4	0.79	0.02	26.78	<0.001
Cynicism				
KBOSS_Q5	0.79	0.03	25.12	<0.001
KBOSS_Q6	0.78	0.02	26.45	<0.001
KBOSS_Q7	0.73	0.03	24.38	<0.001
KBOSS_Q8	0.76	0.02	25.89	<0.001
Inefficacy				
KBOSS_Q9	0.71	0.03	23.67	<0.001
KBOSS_Q10	0.74	0.03	24.91	<0.001
KBOSS_Q11	0.77	0.02	26.23	<0.001
KBOSS_Q12	0.72	0.03	24.18	<0.001

CFI – comparative fit index; Q – question (individual survey item); RMSEA – root mean square error of approximation; SRMR – standardized root mean square residual; TLI – Tucker-Lewis index.

a Model fit indices: χ^2^(246) = 895.42, p < 0.001, CFI = 0.941, TLI = 0.932, RMSEA = 0.045 (90% CI: 0.042–0.048), SRMR = 0.043.

b Model fit indices: χ^2^(51) = 285.36, p < 0.001, CFI = 0.971, TLI = 0.963, RMSEA = 0.038 (90% CI: 0.034–0.042), SRMR = 0.032.

**Table 3.** Comparison of model fit indices among workers in Korean small- and medium-sized enterprises (N = 1024), May 2023 – January 2025

Fit index	χ^2^/df	CFI	TLI	RMSEA	SRMR
Recommended criteria	<3.0	>0.90	>0.90	<0.08	<0.08
Model					
KOSS	3.64	0.941	0.932	0.045	0.043
KBOSS	5.94	0.971	0.963	0.038	0.032
Judgment	acceptable/borderline	excellent	excellent	excellent	excellent

Abbreviations as in Table 2.

### General characteristics

Table 4 presents the general characteristics and BOS of the study participants. The age of all participants was M±SD 42.10±11.86 years. Of the 1024 individuals, 34 (3.3%) were diagnosed with BOS based on the KBOSS criteria. There were more males (N = 826, 80.7%) compared with females (N = 198, 19.3%). A total of 517 (50.5%) participants were married. Among all the workers, 713 (69.6%) were engaged in shift work. When categorized by work type, the majority were employed in production jobs (N = 648, 63.3%), followed by office jobs (N = 274, 26.8%), and service jobs (N = 102, 10.0%). Regarding the length of service, 263 (25.7%) had <1 year of experience, 315 (30.8%) had worked for 1–5 years, 188 (18.4%) for 5–10 years, and 258 (25.2%) for >10 years. Only the work type showed statistically significant association with BOS (p = 0.001).

**Table 4.** General characteristics and burnout syndrome (BOS) among workers at small- and medium-sized enterprises, South Korea, May 2023 – January 2025

Variable	Participants (N = 1024) [n (%)]		M±SD	p^a^
	total	diagnosed BOS (N = 34, 3.3%)		
Age [years]			42.10±11.86	0.554
Sex				0.332
male	826 (80.7)	25 (3.0)		
female	198 (19.3)	9 (4.5)		
Length of service				0.292
<1 year	263 (25.7)	8 (3.0)		
1 to <5 years	315 (30.8)	13 (4.1)		
5 to <10 years	188 (18.4)	8 (4.3)		
≥10 years	258 (25.2)	5 (1.9)		
Shift work status				0.739
yes	713 (69.6)	24 (3.4)		
no	311 (30.4)	10 (3.2)		
Marital status				0.083
married	517 (50.5)	12 (2.3)		
not married	507 (49.5)	22 (4.3)		
Work type				0.001
office worker	274 (26.8)	9 (3.3)		
production worker	648 (63.3)	18 (2.8)		
service worker	102 (10.0)	7 (6.9)		

a p-values were obtained for the diagnosed BOS groups using the χ^2^ test, t-test.

The burnout prevalence of 3.3% in this study population represents a significant occupational health concern, particularly considering that this was identified using stringent diagnostic criteria requiring all 3 burnout dimensions (exhaustion, cynicism, and inefficacy) to exceed their respective cutoff scores simultaneously.

### Distribution of burnout scores among study participants

Table 5 presents the results of the burnout scales for the study participants using KBOSS. According to the KBOSS criteria, exhaustion was considered to exceed the cutoff score if it was ≥21 pts, cynicism if it was ≥18 pts, and inefficacy if it was ≥15 pts. Burnout syndrome is diagnosed when all 3 domains exceed their respective cutoff scores [13].

**Table 5.** Distribution of burnout measurement scales among workers at small- and medium-sized enterprises, South Korea, May 2023–January 2025

Burnout dimension	Score [pts] (M±SD)	Cut-off score	Participants (N = 1024) [n (%)]	
			below cut-off	above cut-off
Exhaustion	15.00±5.39	≥21	883 (86.2)	141 (13.8)
Cynicism	12.12±4.49	≥18	935 (91.3)	89 (8.7)
Inefficacy	12.72±3.69	≥15	710 (69.3)	314 (30.7)
Burnout syndrome^a^		all 3	990 (96.7)	34 (3.3)

a Burnout syndrome meets cut-off criteria for all 3 dimensions simultaneously.

The average scores of the participants were as follows: exhaustion scored M±SD 15.00±5.39 pts, cynicism scored 12.12±4.49 pts, and inefficacy scored 12.72±3.69 pts. The proportion of individuals exceeding the cutoff scores in each domain was 141 (13.8%) for exhaustion, 89 (8.7%) for cynicism, and 314 (30.7%) for inefficacy, respectively. Thirty-four participants (3.3%) met the cutoff criteria for all 3 domains, accounting for the diagnosed BOS cases in the entire study population.

### Burnout syndrome risk based on job stress of study participants

Table 6 evaluates the risk of job stress factors for individuals diagnosed with BOS who met the cutoff scores for all 3 dimensions of burnout. Significant associations were observed when job demands were in Q3 (OR = 12.62, 95% CI: 2.03–78.41, p < 0.05) and when overall job stress was in Q4 (OR = 17.56, 95% CI: 1.40–220.76, p < 0.05). In other job stress domains, the risk of BOS was not statistically significant.

**Table 6.** Association between factors of job stress and burnout syndrome among workers at small- and medium-sized enterprises (N = 1024), South Korea, May 2023 – January 2025

Job stress factor	Burnout syndrome	
	OR (95% CI)	p
Job demand		
Q1	1.00 (ref.)	
Q2	3.56 (0.68–18.73)	0.275
Q3	12.62 (2.03–78.41)	**0.011**
Q4	3.126 (0.69–14.27)	0.283
Job autonomy		
Q1	1.00 (ref.)	
Q2	0.45 (0.09–2.22)	0.326
Q3	0.46 (0.09–2.28)	0.341
Q4	0.48 (0.11–2.16)	0.341
Relationship conflict		
Q1 and Q2	1.00 (ref.)	
Q3	1.56 (0.34–7.12)	0.566
Q4	0.98 (0.21–4.48)	0.976
Job instability		
Q1	1.00 (ref.)	
Q2	0.69 (0.12–3.85)	0.667
Q3	0.71 (0.07–7.60)	0.778
Q4	1.21 (0.21–7.03)	0.834
Organizational system		
Q1	1.00 (ref.)	
Q2	0.41 (0.04–3.87)	0.436
Q3	0.46 (0.04–4.96)	0.525
Q4	0.26 (0.02–2.81)	0.267
Q1 and Q2	1.00 (ref.)	
Inadequate compensation		
Q3	1.44 (0.19–10.86)	0.474
Q4	4.03 (0.84–30.24)	0.069
Work culture		
Q1	1.00 (ref.)	
Q2	0.55 (0.09–3.24)	0.509
Q3	1.11 (0.24–5.16)	0.893
Q4	2.07 (0.40–10.73)	0.387
Job stress		
Q1	1.00 (ref.)	
Q2	2.88 (0.20–41.77)	0.405
Q3	9.51 (0.78–116.01)	0.077
Q4	17.58 (1.40–220.76)	**0.029**

Bold values indicate statistical significance (p < 0.05).

Odds ratios were calculated by multiple logistic regression analysis after adjusting for gender and age, marital status, shift work, work type, and length of service.

#### Hypothesis testing overview

The H1 was supported, with job stress factors showing significant positive associations with burnout dimensions. The H2 and H3 were supported, as job demands showed strong associations with exhaustion (Q3: OR = 12.62, p < 0.05) and organizational factors (inadequate compensation) demonstrated significant relationships with cynicism (Q4: OR = 4.14, p < 0.01), consistent with the hypothesized directions. However, formal statistical comparisons of association strengths were not conducted. The H4 was partially supported, with job autonomy showing significant associations with inefficacy. The H5 was confirmed, with overall job stress demonstrating substantial association with BOS (Q4: OR = 17.58, p < 0.05).

### Association between job stress factors and the 3 dimensions of burnout

Table 7 shows the effects of job stress factors on the 3 burnout dimensions. The criteria for categorizing the quartiles of each job stress factor were based on the Development and Standardization Study of the Korean Job Stress Measurement Tool [14]. For the relationship conflict and inadequate compensation domains, Q1 and Q2 were combined and used as the reference group for analysis due to insufficient sample sizes in Q1.

**Table 7.** Association between dimensions of burnout and job stress among workers at small- and medium-sized enterprises (N = 1024), South Korea, May 2023 – January 2025

Job stress factor	Exhaustion		Cynicism		Inefficacy	
	OR (95% CI)	p	OR (95% CI)	p	OR (95% CI)	p
Job demand						
Q1	1.00 (ref.)		1.00 (ref.)		1.00 (ref.)	
Q2	1.096 (0.47–2.57)	0.476	0.61 (0.23–1.58)	0.304	0.80 (0.52–1.26)	0.353
Q3	10.71 (3.64–31.48)	**0.000**	1.37 (0.39–4.80)	0.619	0.82 (0.34–2.01)	0.666
Q4	3.90 (1.90–7.97)	**0.000**	1.02 (0.46–2.27)	0.960	0.93 (0.57–1.51)	0.766
Job autonomy						
Q1	1.00 (ref.)		1.00 (ref.)		1.00 (ref.)	
Q2	0.43 (0.22–0.85)	0.028	0.62 (0.22–1.71)	0.355	1.66 (0.95–2.89)	0.092
Q3	0.28 (0.13–0.61)	**0.002**	0.83 (0.31–2.24)	0.714	2.78 (1.61–4.82)	**0.000**
Q4	0.23 (0.11–0.48)	**0.000**	1.38 (0.56–3.40)	0.490	3.82 (2.31–6.31)	**0.000**
Relationship conflict						
Q1 and Q2	1.00 (ref.)		1.00 (ref.)		1.00 (ref.)	
Q3	0.36 (0.18–0.70)	**0.006**	0.48 (0.23–1.02)	0.192	1.96 (1.25–3.06)	**0.003**
Q4	0.39 (0.19–0.80)	**0.018**	0.36 (0.17–0.78)	**0.032**	1.98 (1.20–3.27)	**0.007**
Job instability						
Q1	1.00 (ref.)		1.00 (ref.)		1.00 (ref.)	
Q2	0.60 (0.27–1.34)	0.178	1.05 (0.37–2.98)	0.922	1.62 (0.94–2.77)	0.113
Q3	0.82 (0.29–2.28)	0.232	1.84 (0.42–8.06)	0.418	2.78 (1.48–5.23)	**0.036**
Q4	1.28 (0.53–3.09)	0.875	1.67 (0.54–5.14)	0.370	1.82 (0.96–3.44)	0.053
Organizational system						
Q1	1.00 (ref.)		1.00 (ref.)		1.00 (ref.)	
Q2	0.68 (0.30–1.54)	0.359	1.24 (0.39–3.94)	0.712	1.21 (0.78–1.87)	0.406
Q3	0.53 (0.20–1.41)	0.203	1.44 (0.39–5.30)	0.583	1.17 (0.63–2.19)	0.620
Q4	0.86 (0.33–2.23)	0.755	0.86 (0.23–3.18)	0.812	1.28 (0.67–2.47)	0.456
Inadequate compensation						
Q1 and Q2	1.00 (ref.)		1.00 (ref.)		1.00 (ref.)	
Q3	0.91 (0.44–1.90)	0.835	1.88 (0.69–6.94)	0.198	1.83 (1.22–2.73)	**0.033**
Q4	2.06 (1.02–4.16)	**0.050**	4.14 (1.60–10.53)	**0.001**	2.85 (1.90–4.25)	**0.000**
Work culture						
Q1	1.00 (ref.)		1.00 (ref.)		1.00 (ref.)	
Q2	0.94 (0.44–2.02)	0.970	0.56 (0.21–1.45)	0.229	1.66 (1.06–2.61)	**0.029**
Q3	1.51 (0.70–3.25)	0.286	0.89 (0.36–2.20)	0.793	2.11 (1.31–3.41)	**0.004**
Q4	2.63 (1.11–6.24)	**0.030**	1.66 (0.61–4.50)	0.317	2.33 (1.30–4.16)	**0.005**
Job stress						
Q1	1.00 (ref.)		1.00 (ref.)		1.00 (ref.)	
Q2	3.28 (1.26–8.57)	**0.009**	2.10 (0.64–6.94)	0.604	1.17 (0.66–2.06)	0.600
Q3	6.57 (2.32–18.58)	**0.000**	6.39 (2.14–19.05)	**0.046**	1.08 (0.50–2.34)	0.839
Q4	8.63 (2.30–32.44)	**0.000**	17.48 (5.59–54.62)	**0.025**	0.69 (0.23–1.95)	0.466

Bold values indicate statistical significance (p < 0.05).

Odds ratios were calculated by multiple logistic regression analysis after adjusting for gender and age, marital status, shift work, work type, and length of service.

#### Exhaustion dimension

Statistically significant results indicated that in the exhaustion dimension, compared with Q1 (lowest job demand), the OR increased to 10.71 (95% CI: 3.64–31.48, p < 0.001) in Q3 and 3.90 (95% CI: 1.90–7.97, p < 0.001) in Q4. In the inadequate compensation category, the OR of the Q4 group was 2.06 (95% CI: 1.02–4.16, p < 0.05), which was higher than that of the Q1 and Q2 group. Additionally, compared with Q1, where workplace culture was perceived as the best, the OR in Q4 increased to 2.63 (95% CI: 1.11–6.24, p < 0.05). Furthermore, compared with Q1 where overall job stress was the lowest, the OR values for Q2, Q3, and Q4 increased to 3.28 (95% CI: 1.26–8.57, p < 0.01), 6.57 (95% CI: 2.32–18.58, p < 0.001), and 8.63 (95% CI: 2.30–32.44, p < 0.001), respectively.

In contrast, job autonomy showed protective effects with decreased ORs in Q2 (0.43, 95% CI: 0.22–0.85, p < 0.05), Q3 (0.28, 95% CI: 0.13–0.61, p < 0.01), and Q4 (0.23, 95% CI: 0.11–0.48, p < 0.001) compared with Q1. Regarding relationship conflict, the ORs were significantly lower in Q3 (0.36, 95% CI: 0.18–0.70, p < 0.01) and Q4 (0.39, 95% CI: 0.19–0.80, p < 0.05) compared with Q1 and Q2.

#### Cynicism dimension

In the cynicism dimension, compared with Q1 and Q2 where compensation was perceived as adequate, the OR value in Q4 was 4.11 (95% CI: 1.60–10.53, p < 0.01). Additionally, the overall job stress score showed significant increases with ORs of 6.39 (95% CI: 2.14–19.05, p < 0.05) in Q3 and 17.48 (95% CI: 5.59–54.62, p < 0.05) in Q4 compared with Q1. Relationship conflict showed protective effects with significantly decreased ORs of 0.36 (95% CI: 0.17–0.78, p < 0.05) in Q4 compared with Q1 and Q2.

#### Inefficacy dimension

In the inefficacy dimension, a consistent pattern was observed where the risk of inefficacy increased as job stress increased. Compared with Q1 where job autonomy was the highest, the risk significantly increased to 2.78 (95% CI: 1.61–4.82, p < 0.001) in Q3 and 3.82 (95% CI: 2.31–6.31, p < 0.001) in Q4. In the relationship conflict area, compared with Q1 and Q2, the risk increased to 1.96 (95% CI: 1.25–3.08, p < 0.01) in Q3 and 1.98 (95% CI: 1.20–3.27, p < 0.01) in Q4. For job instability, compared with Q1, the risk significantly increased to 2.78 (95% CI: 1.48–5.23, p < 0.005) in Q3. Regarding inadequate compensation, compared with Q1 and Q2, the risk increased to 1.83 (95% CI: 1.22–2.73, p < 0.05) in Q3 and 2.85 (95% CI: 1.90–4.25, p < 0.001) in Q4. In the workplace culture area, the risk increased to 1.66 (95% CI: 1.06–2.61, p < 0.05) in Q2, 2.11 (95% CI: 1.31–3.41, p < 0.01) in Q3, and 2.33 (95% CI: 1.30–4.16, p < 0.01) in Q4 compared with Q1.

## DISCUSSION

This study examined the multidimensional relationship between job stress factors and burnout among Korean workers in small- and medium-sized enterprises, utilizing validated Korean instruments (KOSS and KBOSS) across diverse occupational groups. The research tested 5 specific hypotheses regarding the differential relationships between job stress domains and burnout dimensions, providing both confirmatory and surprising findings that advance the authors' understanding of occupational stress dynamics in Korean workplace contexts.

### Hypothesis testing and primary findings

This study's findings provided strong support for H1, confirming that job stress factors showed significant positive correlations with burnout dimensions, consistent with recent meta-analysis demonstrating the persistent relationship between workplace stressors and burnout across diverse populations [18]. The confirmation of H5 was particularly noteworthy, as the combined effect of multiple job stress factors explained substantial variance in BOS, aligning with contemporary research emphasizing the cumulative impact of multiple stressors in modern work environments [19].

Consistent with established literature and the job demands-resources (JD-R) model, the authors' findings confirmed that high job demands and overall job stress significantly increased burnout risk, supporting theoretical predictions [20,21]. Specifically, moderate-to-high job demands (Q3) and severe overall job stress (Q4) emerged as the strongest predictors of BOS. This aligns with updated JD-R model applications showing that psychological and physical resource depletion from excessive job demands creates a pathway to burnout, particularly through emotional exhaustion and time pressure [22]. The dose-response relationship observed between job demands and burnout risk supports recent theoretical developments emphasizing that sustained high demands without adequate resources lead to progressive energy depletion and self-regulation failure [23].

Inadequate compensation consistently increased risk across multiple burnout dimensions (exhaustion Q4, cynicism Q4, inefficiency Q3–Q4), providing support for H3 regarding organizational factors. This finding reinforces the importance of fair reward systems in preventing burnout and aligns with recent research on effort-reward imbalance as a critical predictor of occupational stress [24]. Similarly, poor workplace culture significantly contributed to both exhaustion (Q4) and inefficiency risks (Q2–Q4), highlighting the critical role of organizational climate in employee wellbeing. These findings support recent studies emphasizing the protective effects of adequate organizational support and fair treatment in mitigating burnout risk, with contemporary research showing that positive organizational climates serve as crucial job resources in the workplace [25,26].

### Unexpected findings and theoretical implications

The results showed significant associations between job demands and exhaustion, and between organizational factors (inadequate compensation) and cynicism, which is consistent with the broad direction of H2 and H3. However, without direct statistical comparison of association strengths, it cannot be definitively concluded that these relationships are stronger than others. Therefore, H2 and H3 are only partially supported by this study's data. Similarly, H4 regarding job insecurity and reduced efficacy showed mixed support, with some significant associations observed but without comparative strength analysis. Contrary to the authors' predictions and previous studies [27,28], the results revealed that decreased job autonomy and increased relationship conflict were associated with reduced risk in certain burnout dimensions. These counterintuitive findings require careful interpretation and should be considered speculative pending replication, as they contradict recent systematic reviews confirming the protective effects of job control and positive interpersonal relationships [29]. It should be noted that the analysis examined individual associations between job stress factors and burnout dimensions but did not include formal statistical comparisons of correlation strengths across different stress-burnout pathways. This represents a limitation in the authors' hypothesis testing approach, particularly for H2 and H3, which implied differential strength of relationships.

For job autonomy, the inverse relationship with exhaustion risk may reflect the “too-much-of-a-good-thing” theory [30], where excessive autonomy creates decision-making burden and role ambiguity that paradoxically increase stress. Recent research has supported this non-linear relationship, suggesting that optimal levels of autonomy exist beyond which additional control becomes counterproductive [31]. When autonomy is constrained, clearer structure and reduced decision-making responsibility may serve as protective factors against exhaustion. Additionally, according to conservation of resources theory [19,20], employees in low-autonomy environments may conserve psychological resources by reducing work investment, thereby protecting against energy depletion. This aligns with recent studies on job crafting, where employees in highly structured environments may experience reduced cognitive load and decision fatigue [41,42].

Contrary to conventional wisdom and previous studies [15,16], this study's results revealed that decreased job autonomy and increased relationship conflict were associated with reduced risk in certain burnout dimensions. These paradoxical findings require careful interpretation and should be considered speculative pending replication. For job autonomy, the inverse relationship with exhaustion risk may reflect the “too-much-of-a-good-thing” theory [17], where excessive autonomy creates decision-making burden and role ambiguity that paradoxically increase stress. When autonomy is constrained, clearer structure and reduced decision-making responsibility may serve as protective factors against exhaustion. Additionally, according to conservation of resources theory [32], employees in low-autonomy environments may conserve psychological resources by reducing work investment, thereby protecting against energy depletion.

The protective effect of relationship conflict against exhaustion and cynicism presents a more complex phenomenon that challenges H2 predictions. Several speculative mechanisms may explain this finding, supported by emerging research on positive conflict outcomes. Conflict resolution processes may strengthen team cohesion and communication skills [33], while organizations tolerating open conflict expression may provide healthier emotional outlets than those requiring conflict suppression [34]. Recent studies on psychological safety suggest that workplaces allowing constructive conflict may foster better team performance and reduced emotional exhaustion [35]. Moderate interpersonal tension may prevent the detachment characteristic of burnout by maintaining active social engagement [36] and conflict-rich environments may paradoxically offer stronger social support networks as protective factors.

Contemporary research on team dynamics indicates that moderate task conflict can enhance problem-solving capabilities and prevent cynicism through increased engagement [37]. However, these interpretations are speculative and may reflect cultural factors specific to Korean workplace dynamics, where hierarchical structures and collectivistic values may moderate conflict-burnout relationships differently than in Western contexts. The cross-sectional design precludes causal inference, and these counterintuitive findings require longitudinal validation and cultural context consideration.

### Practical implications and contemporary relevance

The multidimensional analysis reveals that different job stressors affect distinct burnout components, suggesting targeted intervention strategies particularly relevant in the workplace. For exhaustion prevention, organizations should prioritize workload management and compensation adequacy, aligning with recent findings that work-life balance initiatives have become critical for employee retention [38]. For cynicism reduction, fair reward systems appear most critical, supporting contemporary research on the importance of recognition and career development in maintaining employee engagement [39]. For inefficiency prevention, a broader approach addressing job stability, compensation, and workplace culture is indicated, consistent with recent studies emphasizing the multifaceted nature of modern workplace wellbeing interventions [40]. The finding that relationship conflict may have protective effects in Korean organizational contexts suggests that conflict management training, rather than conflict avoidance, might be more beneficial. However, this recommendation requires further validation given the speculative nature of the authors' interpretation and the need for cultural adaptation of workplace interventions.

### Study strengths and limitations

This study's primary strength lies in its use of ICD-11 aligned burnout assessment tools specifically validated for Korean workers, providing culturally appropriate measurement across diverse occupational groups rather than single-occupation studies. The multidimensional approach to both job stress and burnout components offers nuanced insights for targeted interventions, particularly relevant given recent calls for more sophisticated burnout measurement approaches. Also, the comprehensive psychometric validation through confirmatory factor analysis strengthens confidence in the authors' findings. Both KOSS and KBOSS demonstrated robust factor structures consistent with their theoretical foundations, with the KBOSS showing particularly excellent model fit indices. This validation is crucial given the cultural adaptation of these instruments for Korean workers and supports the reliability of the multidimensional burnout findings.

However, several limitations must be acknowledged. The cross-sectional design prevents determination of causal relationships between job stressors and burnout dimensions. The relatively low prevalence of cynicism (8.7%) resulted in wide confidence intervals, limiting statistical power and the precision of the estimated associations for this dimension. This should be considered when interpreting the findings related to cynicism. Findings from Korean SME workers may not directly apply to other cultural contexts or organizational types, highlighting the need for cross-cultural validation of burnout research. Limited industry-specific analysis may obscure occupation-specific patterns, and the analysis did not fully account for potential interactions between different job stress factors, areas that recent research has identified as critical for understanding contemporary workplace stress. Survey-based assessment may not capture organizational cultural nuances or individual coping mechanisms that qualitative methods might reveal, a limitation increasingly recognized in recent mixed-methods burnout research. Although this study utilized standardized criteria from the KOSHA to enhance practical applicability, future research may require validation through diverse analytical approaches, including digital biomarkers and ecological momentary assessment methods that are emerging in occupational health research. This study did not conduct formal statistical tests to compare the relative strength of associations between different job stressors and burnout dimensions, limiting the authors' ability to make definitive claims about the differential impact of specific stressors. Future research should include statistical comparisons (e.g., Wald tests, effect size comparisons) to rigorously test hypotheses regarding the relative strength of different stress-burnout relationships.

## CONCLUSIONS

This study provides empirically-grounded evidence that job stress factors differentially affect specific burnout dimensions among Korean SME workers, with findings both confirming established theories and revealing unexpected cultural-specific patterns. While the authors' hypotheses regarding the positive relationships between job stressors and burnout were largely supported, the paradoxical findings for job autonomy and relationship conflict highlight the complexity of workplace stress dynamics in Korean organizational contexts. Job demands and overall stress emerge as primary targets for burnout prevention, while adequate compensation and positive workplace culture serve as critical protective factors, findings that are particularly relevant given the increased focus on workplace mental health in the post-pandemic era.

Based on the authors' findings, organizations should implement evidence-based workload management programs focusing on job demands assessment and redistribution, establish fair compensation review systems to address reward inadequacy, and develop workplace culture improvement initiatives emphasizing supportive organizational climate. The unexpected finding that relationship conflict may have protective effects suggests that conflict management training, rather than conflict suppression, might be beneficial, though this requires further validation given the speculative nature of this study's interpretation and the need for cultural adaptation of workplace interventions.

Future investigations should employ longitudinal designs to establish causal relationships and test the authors' hypotheses more rigorously, include protective factors such as resilience and social support alongside risk factors, conduct cross-cultural validation of the paradoxical autonomy and conflict findings, integrate qualitative assessments to understand organizational context effects, and expand sample diversity across industries and organizational sizes. The unexpected findings regarding job autonomy and relationship conflict highlight the complexity of workplace stress dynamics and underscore the need for culturally-informed, multidimensional approaches to occupational health research and intervention development that consider the evolving nature of work in the digital age.
